# Aortic Root Pathologies and Surgical Management: Insights From a Single Surgeon’s Experience

**DOI:** 10.7759/cureus.74096

**Published:** 2024-11-20

**Authors:** Muhammad Aasim, Raheela Aziz, Atta ul Mohsin, Raheel Khan, Gulshad Aziz, Ayesha Zahid, Aariya Srinivasan, Jibran Ikram

**Affiliations:** 1 Cardiac Surgery, Hayatabad Medical Complex, Peshawar, PAK; 2 Cardiac Surgery, Khyber Girls Medical College (KGMC), Peshawar, PAK; 3 Cardiovascular Medicine, Hayatabad Medical Complex, Peshawar, PAK; 4 Anatomy, Khyber Medical University (KMU) Institute of Dental Sciences, Peshawar, PAK; 5 Outcomes Research, Cleveland Clinic, Cleveland, USA

**Keywords:** aortic root dissection, aortic root surgery, ascending aorta replacement, bentall operation, bicuspid aortic valve, marfan syndrome, mini-bentall

## Abstract

Introduction and objectives

The Bentall procedure is a surgical technique designed to address aortic root abnormalities, including issues with the aortic valve, aortic root, and ascending aortic disease. This study aimed to assess the short-term outcomes of 39 patients who underwent the Bentall and concomitant procedures: aortic root enlargement, personalized external aortic root support (PEARS), and Mini-Bentall procedures at a single center.

Methodology

We conducted a retrospective study involving 39 patients who underwent surgery for aortic root pathologies such as dissection, Marfan syndrome (MFS), bicuspid aortic valve, degenerative disease, and atherosclerosis at our hospital between January 2019 and September 2024. Data were collected from clinical records and were utilized for statistical analysis.

Results

In this study of 39 patients (average age 43.97 ± 17.45 years; 71.8% male), hypertension was the most common risk factor (46.2%). The early mortality rate was 2.6%, with one death from bleeding. Dissection and MFS were the leading causes of aortic root pathologies (35.9% each). Coexisting heart diseases were found in 20.5% of patients. Emergency and urgent surgeries accounted for 38.5% and 53.8%, respectively. Bentall surgery was performed in 64.4% of cases, with average cardiopulmonary bypass (CPB) and cross-clamp times of 196.10 ± 25.23 and 169.05 ± 23.9 minutes, respectively.

Conclusion

Overall, the hospital mortality rate for the Bentall procedure at our institution was 2.6%, consistent with the reported literature. Our results show that, although complex, the classic Bentall technique can be performed safely with acceptable short-term morbidity and mortality. Addressing complications like hospital mortality and postoperative bleeding is crucial, as these issues may be preventable.

## Introduction

Aortic root replacement with reattachment of the two main coronary arteries was originally described by Bentall and De Bono in 1968 [1]. Now, this well-documented technique of aortic root replacement has been used for a large spectrum of various pathologic conditions involving the aortic valve, aortic root, and ascending aorta [2-4]. Although the operation was originally designed to treat patients with aortic root aneurysms, the indications for radical root replacement have expanded [5-7]. An ascending aortic aneurysm typically develops slowly and remains asymptomatic until it causes severe complications, such as rupture or dissection. When rupture or dissection occurs, many patients experience fatal outcomes before reaching the hospital. Management of thoracic aortic conditions is challenging in both elective and emergency cases, with decisions about when to operate often weighing surgical risks against rupture risk in elective cases, while mortality in cases of thoracic aortic rupture is extremely high, ranging from 94% to 100% [8].

In a population-based study done by Clouse et al., the five-year rupture risk for aneurysms was 0% for those less than 4 cm, 16% (95% CI: 4%-28%) for those 4-5.9 cm, and 31% (95% CI: 5%-56%) for those 6 cm or larger [9]. For those who do make it to the hospital, emergency surgery is required, which carries a mortality risk of about 20%. In contrast, elective replacement of the ascending aorta has a much lower risk of both mortality and morbidity [10]. The following were identified as independent risk factors for rupture: old age, pain, chronic obstructive pulmonary disease, and maximal identified thoracic and abdominal aortic aneurysm [11].

Also, dissection and rupture of the aortic root are the leading causes of death among patients with Marfan syndrome (MFS) [12,13]. MFS is due to genetic mutations in the FBN1 gene that lead to abnormal production and cellular handling of the fibrillin-1 protein [14]. Before the advent of surgical intervention in these patients, one series reported a mean survival of about 44 years, and most premature deaths were due to cardiovascular causes [15]. Survival in patients with MFS has been improved by the introduction of prophylactic surgical aortic root replacement [16]. Over the years, this procedure underwent important modifications, including the abandonment of the wrap-inclusion technique in favor of the coronary-button technique, and it has become a widely adopted standard of treatment for various root pathologies. In fact, although valve-sparing procedures are often the preferred approach in selected cases, the Bentall operation remains universally applicable in an all-comer cohort and is not limited to favorable valve morphology, including patients at high risk of aortic complications, i.e., genetic family syndromes, bicuspid valve, altered root geometry, and coronary ostia dislocation. Complete aortic root replacement can be performed using biological or mechanical valved conduits [17-19].

## Materials and methods

This retrospective study was performed at the Cardiac Surgery Department of Hayatabad Medical Complex, Peshawar, Pakistan. The study included 39 patients in whom aortic root reconstruction for a large spectrum of various pathologic conditions involving the aortic valve, aortic root, and ascending aorta was performed in 2018. We started Mini-Bentall procedures in July 2024 at our center. All patients underwent a preoperative transthoracic echocardiogram and CT-aortogram to assess aortic diameter and valve function. Variables collected on patients included demographic and comorbidity information, echocardiographic parameters (including degree of aortic insufficiency (AI)), operative data, and postoperative data (including complications and mortality). Descriptive statistics were used; continuous variables are presented as means and standard deviations, and categorical variables are summarized as frequencies and percentages. Analysis was performed using IBM SPSS Statistics for Windows, Version 20 (Released 2011; IBM Corp., Armonk, NY, USA).

Aim

The aim of our study is to assess the short-term outcomes of aortic root reconstruction for a broad range of pathological conditions involving the aortic valve, aortic root, and ascending aorta at our center since 2018.

Surgical technique

The Bentall procedure was performed in all patients through a median sternotomy, except for two patients in whom an upper J-shaped mini-sternotomy was used. Cardiopulmonary bypass (CPB) was established via femoral artery cannulation in 36 patients (92.3%) and aortic cannulation in three patients (7.7%), with right atrial cannulation as well. Using moderate systemic hypothermia (30ºC-32ºC), the ascending aorta was clamped, and cardiac arrest was achieved with cold hyperkalemic crystalloid cardioplegia and local hypothermia.

Aneurysm identification was accomplished through longitudinal aortotomy, followed by resection of the native aortic valve. Anastomosis of the valved tubular graft to the aortic valve ring was performed using a 2-0 continuous polypropylene suture. Coronary ostia were implanted through two openings in the graft using a 5-0 polypropylene suture. If the aneurysm extended to the ascending aorta without dissection, the graft was sized, and distal anastomosis to the aorta was completed. In cases of dissection, a modified technique was used, involving Teflon material to plicate the aortic wall and graft to obliterate the false lumen and reinforce the anastomosis.

The rewarming phase was initiated, with the aortic root and left chambers purged via the aortic root and right upper pulmonary vein. After completing both the distal and proximal anastomoses, the residual aortic wall was sutured around the tubular graft. The procedure was completed in the conventional manner.

Postoperative care

All patients undergoing valve-sparing aortic root replacement (VSRR) received aspirin therapy for one month postoperatively, as well as beta-blocker therapy indefinitely. A pre-discharge echocardiogram was performed, and the patient was then advised to follow up after one week, then after one month, then after three months with an echocardiogram, then after six months with an echocardiogram, and then annually; follow-up exams were recommended. Patients who received Bentall procedures required lifelong warfarin therapy. The target international normalized ratio was 2.0 to 3.5. Post-discharge care was directed by the patient’s referring cardiologist.

## Results

**Figure 1:**
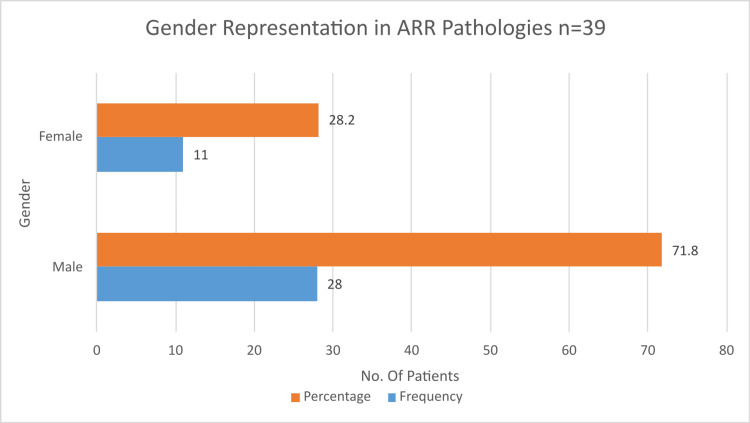
Gender representation in aortic root replacement (N = 39) ARR: Aortic root replacement

A total of 39 patients were included in the final analysis. Preoperative and demographic details were recorded. The average age was 43.97 ± 17.45 years, with a predominance of males (28 patients, or 71.8%) (Figure 1).

**Figure 2:**
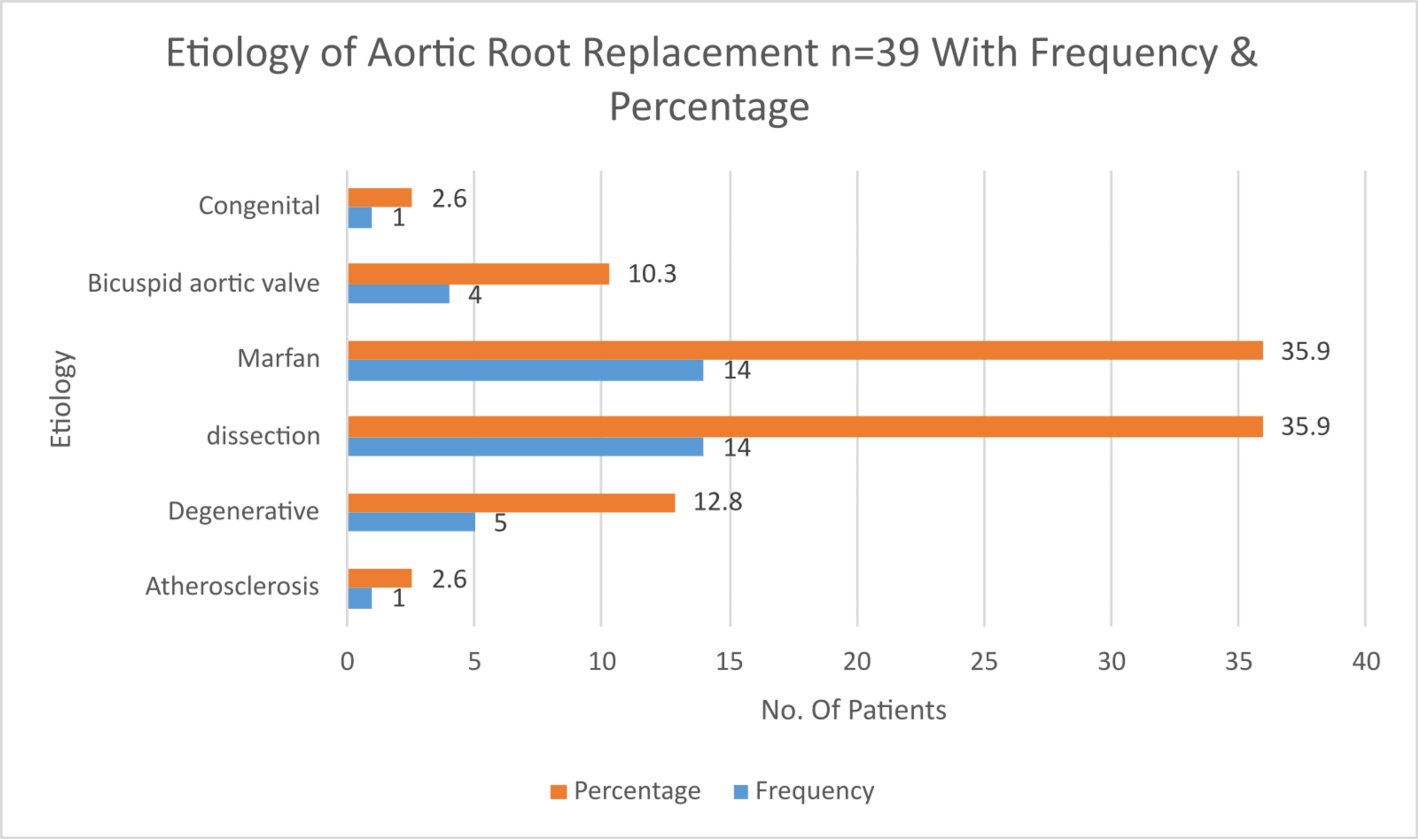
Etiology of aortic root replacement (n = 39) with frequency and percentage

Hypertension was the most common risk factor (n = 18, or 46.2%). The mortality rate was 2.6%. The rate of death within 30 days of the initial hospitalization, referred to as the overall early mortality rate, was 2.6%, with 1 out of 39 patients affected. The cause of early death in that one patient was excessive bleeding. Early complications included re-exploration for bleeding in two patients (5.1%). The most common causes were dissection and MFS associated with valvular disease, each accounting for 35.9% of cases. Atherosclerosis was identified in 2.6% of cases. The bicuspid aortic valve was present in 10.3% of cases, while genetic or congenital diseases, like Williams syndrome, were observed in 2.6% of cases. Degenerative conditions were found in 12.8% of cases. Heart disease coexisting with aortic root pathologies was seen in eight patients (20.5%). This included ischemic heart disease in five patients, severe mitral insufficiency in one patient, and moderate mitral insufficiency in two patients. Emergency surgery was required in 15 cases (38.5%), urgent surgery in 21 cases (53.8%), with aneurysmal size being the primary indication. Elective surgery was performed in three cases (7.7%). For aortic root pathologies treatment, Bentall surgery was performed in 25 cases (64.4%). Aortic root enlargement was carried out in two patients (5.1%), while in one patient, the personalized external aortic root support (PEARS) was carried out (2.1%). Additionally, eight cases (20.5%) involved concomitant procedures, such as myocardial revascularization in five cases, mitral valve implantation in one case, and mitral valve repair in two cases (Table 1; Figure 2).

**Table 1:** Demographic and clinical characteristics CABG: Coronary artery bypass graft; MVR: Mitral valve replacement; MV: Mitral valve; CAD: Coronary artery disease; DM: Diabetes mellitus; VSRR: Valve-sparing aortic root replacement

Parameters	Frequency	Percentage
Male	28	71.8
Female	11	28.2
Hypertension	18	46.2
Smoking	5	12.8
DM	7	17.9
CAD single vessel	3	7.7
CAD double vessel	1	2.6
CAD triple vessel	1	2.6
Previous cardiac surgery	0	0
Cardiovascular accident	0	0
Reintubation	0	0
Re-exploration	2	5.1
In-hospital mortality	1	2.6
Surgery performed: Bentall	25	64.4
Bentall + CABG	5	12.8
Bentall + MVR	1	2.6
Bentall + MV repair	2	5.1
Mini Bentall	2	5.1
Canulation: Femoral	36	92.3
Aortic	3	7.7
VSRR	1	2.6
Others	3	7.7
Aortic regurgitation	13	33.3
Moderate	9	23.1
Severe	17	43.6
Aortic stenosis mild	7	17.9
Moderate	4	10.3
Severe	8	20.5
Etiology division		
Atherosclerosis	1	2.6
Degenerative	5	12.8
Dissection	14	35.9
Marfan	14	35.9
Bicuspid aortic valve	4	10.3
Congenital	1	2.6
Timing of operation		
Emergent	15	38.5
Urgent	21	53.8
Elective	3	7.7
Blood transfusion		
None transfused	4	10.3
500 mL transfused	14	35.9
1000 mL transfused	13	33.3
1500 mL transfused	3	7.7
2000 mL transfused	3	7.7
More than 2000 mL	2	5.1

Average CPB and cross-clamp times were 196.10 ± 25.23 minutes and 169.05 ± 23.9 minutes, respectively (Table 2).

**Table 2:** Demographic and intraoperative parameters of patients undergoing aortic root surgery CPB: Cardiopulmonary bypass; ICU: Intensive care unit

Parameters	Mean ± SD	Min	Max
Age (years)	43.97 ± 17.45	11	70
CBP time (minutes)	196.10 ± 25.23	126	248
X-clamp time (minutes)	169.05 ± 23.9	101	212
Drainage (mL)	934.62 ± 526.4	350	2650
ICU stay in hours	47.72 ± 11.05	40	91
Ventilation time in hours	8.64 ± 4.53	4	22

## Discussion

Nearly six decades have passed since Bentall and De Bono [1] introduced a pioneering surgical technique for managing aortic root aneurysms. Over time, this technique has evolved into the gold standard for treating abnormalities of the aortic valve, ascending aorta, and aortic root. Despite advancements, the procedure remains intricate and demanding, especially in emergencies like acute aortic dissection. When an aortic valve is damaged and there is aortic root dilation or dissection, the Bentall procedure becomes essential. There is ongoing debate about whether valve-sparing techniques are appropriate only for selected patients, or if they have broader applicability, and whether their long-term durability exceeds that of valve replacement. As a result, the modified Bentall procedure is anticipated to remain the standard approach for managing various aortic root conditions.

This study represents a single-center experience involving patients with different aortic root pathologies, such as ascending aortic dissection and/or dilated ascending aorta. We observed that the hospital mortality rate for patients undergoing the Bentall procedure to treat various aortic root pathologies was 2.6%, with excessive bleeding being the cause. The low operative mortality rate of 2.6% may be attributed to various intraoperative technical factors that we employed. The most concerning complications following a Bentall procedure are associated with improper positioning of the perianastomotic suture of the coronary ostia. Such malposition can lead to torsion, dissection, laceration, or the late formation of a pseudoaneurysm. Incorrect reimplantation of the ostia may result in severe outcomes, including myocardial infarction, low cardiac output syndrome, and right ventricular dysfunction, potentially leading to mortality.

We chose to use mechanical prostheses for all our patients who did not have contraindications to long-term anticoagulation. This approach is based on the observation from the most recent 2021 guidelines, which indicated higher mortality rates during long-term follow-up (at 10 and 15 years) for patients under 60 years old, and those between 50 and 70 years old who had a biological valve implanted in the aortic position [20]. The mean duration of CPB was 196.10 ± 25.23 minutes, and aortic clamping was 169.05 ± 23.9 minutes, as observed in our study. In this study, femoral arterial cannulation was employed for all cases of aortic dissection to facilitate the initiation of antegrade cerebral perfusion during circulatory arrest. Additionally, to decrease myocardial oxygen demand and work in a bloodless field for enhanced myocardial protection, an antegrade del Nido cardioplegic solution was administered through the ostial cannula. In this study, postoperative bleeding occurred in two patients (5.1%), necessitating re-exploration. However, Zehr et al. reported that eight cases (4%) experienced bleeding that required re-exploration [21]. Additionally, Etz et al. documented a reoperation rate of 18.3% due to bleeding [22]. In this study, no re-explorations were needed due to the failure of the Bentall procedure, and the outcomes following the Bentall procedure were satisfactory. These results align with those reported by Yakut [23].

Limitations

The study's limitations include its retrospective design, which may introduce bias, and a small sample size of only 39 patients, limiting generalizability. Conducted at a single center, the findings may not reflect broader practice variations. Additionally, the short follow-up period restricts the assessment of long-term outcomes and complications.

## Conclusions

In conclusion, the hospital mortality rate for the Bentall procedure to treat ascending aortic aneurysmal disease with aortic valve replacement at our institution was 2.6%, aligning with rates reported in the literature. Our findings demonstrate that, despite its complexity and relative infrequency, the classic Bentall technique for managing ascending aortic aneurysms and dissection can be safely performed with acceptable short-term morbidity and mortality at our center. The Bentall procedure is confirmed as a landmark treatment for aortic root and ascending aorta pathologies. Complications like hospital mortality and postoperative bleeding underscore the practical importance of addressing potentially preventable issues in this high-stakes procedure.

## References

[1] Bentall H, De Bono A (1968). A technique for complete replacement of the ascending aorta. Thorax.

[2] Kouchoukos NT, Wareing TH, Murphy SF, Perrillo JB (1991). Sixteen-year experience with aortic root replacement. Results of 172 operations. Ann Surg.

[3] Svensson LG, Crawford ES, Hess KR (1992). Composite valve graft replacement of the proximal aorta: comparison of techniques in 348 patients. Ann Thorac Surg.

[4] Pacini D, Ranocchi F, Angeli E (2003). Aortic root replacement with composite valve graft replacement of the proximal aorta. Ann Thorac Surg.

[5] Etz CD, Homann TM, Silovitz D (2007). Long-term survival after the Bentall procedure in 206 patients with bicuspid aortic valve. Ann Thorac Surg.

[6] Hagl C, Galla JD, Lansman SL (2002). Replacing the ascending aorta and aortic valve for acute prosthetic valve endocarditis: is using prosthetic material contraindicated?. Ann Thorac Surg.

[7] Halstead JC, Spielvogel D, Meier DM (2005). Composite aortic root replacement in acute type A dissection: time to rethink the indications?. Eur J Cardiothorac Surg.

[8] Olsson C, Thelin S, Ståhle E, Ekbom A, Granath F (2006). Thoracic aortic aneurysm and dissection: increasing prevalence and improved outcomes reported in a nationwide population-based study of more than 14,000 cases from 1987 to 2002. Circulation.

[9] Clouse WD, Hallett JW Jr, Schaff HV, Gayari MM, Ilstrup DM, Melton LJ 3rd (1998). Improved prognosis of thoracic aortic aneurysms: a population-based study. JAMA.

[10] Guo MH, Appoo JJ, Saczkowski R (2018). Association of mortality and acute aortic events with ascending aortic aneurysm: a systematic review and meta-analysis. JAMA Netw Open.

[11] Juvonen T, Ergin MA, Galla JD (1997). Prospective study of the natural history of thoracic aortic aneurysms. Ann Thorac Surg.

[12] Judge DP, Dietz HC (2005). Marfan's syndrome. Lancet.

[13] Marsalese DL, Moodie DS, Vacante M (1989). Marfan’s syndrome: natural history and long-term follow-up of cardiovascular involvement. J Am Coll Cardiol.

[14] Fleischer KJ, Nousari HC, Anhalt GJ (1997). Immunohistochemical abnormalities of fibrillin in cardiovascular tissues in Marfan’s syndrome. Ann Thorac Surg.

[15] Murdoch JL, Walker BA, Halpern BL, Kuzma JW, McKusick VA (1972). Life expectancy and causes of death in the Marfan syndrome. N Engl J Med.

[16] Silverman DI, Burton KJ, Gray J (1995). Life expectancy in the Marfan syndrome. Am J Cardiol.

[17] Wallen T, Habertheuer A, Bavaria JE (2019). Elective aortic root replacement in North America: analysis of STS adult cardiac surgery database. Ann Thorac Surg.

[18] Stamou SC, Williams ML, Gunn TM, Hagberg RC, Lobdell KW, Kouchoukos NT (2015). Aortic root surgery in the United States: a report from the society of thoracic surgeons database. J Thorac Cardiovasc Surg.

[19] Nardi P, Pisano C, Ruvolo G (2020). The need for the STS score risk stratification system for aortic root aneurysms surgery. Ann Thorac Surg.

[20] Vahanian A, Beyersdorf F, Praz F (2022). 2021 ESC/EACTS guidelines for the management of valvular heart disease. Eur Heart J.

[21] Zehr KJ, Orszulak TA, Mullany CJ (2004). Surgery for aneurysms of the aortic root: a 30-year experience. Circulation.

[22] Etz CD, Bischoff MS, Bodian C, Roder F, Brenner R, Griepp RB, Di Luozzo G (2010). The Bentall procedure: is it the gold standard? A series of 597 consecutive cases. J Thorac Cardiovasc Surg.

[23] Yakut C (2001). A new modified Bentall procedure: the flanged technique. Ann Thorac Surg.

